# Genetic Characterization of Mutations Related to Conidiophore Stalk Length Development in *Aspergillus niger* Laboratory Strain N402

**DOI:** 10.3389/fgene.2021.666684

**Published:** 2021-04-20

**Authors:** Ebru Demirci, Mark Arentshorst, Baran Yilmaz, Aram Swinkels, Ian D. Reid, Jaap Visser, Adrian Tsang, Arthur F. J. Ram

**Affiliations:** ^1^Institute of Biology Leiden, Microbial Sciences, Leiden University, Leiden, Netherlands; ^2^Centre for Structural and Functional Genomics, Concordia University, Montreal, QC, Canada; ^3^Fungal Genetics and Technology Consultancy, Wageningen, Netherlands

**Keywords:** conidiophore stalk length, radial growth, *Aspergillus niger*, genome sequencing, fungal development, kinesins

## Abstract

*Aspergillus niger* is an important filamentous fungus in industrial biotechnology for the production of citric acid and enzymes. In the late 1980s, the *A. niger* N400/NRRL3 strain was selected for both fundamental and applied studies in relation to several processes including gluconic acid and protein production. To facilitate handling of *A. niger*, the N400 wild-type strain was UV mutagenized in two consecutive rounds to generate N401 and N402. N402 was used as a reference laboratory strain and exhibits the phenotypes with reduced conidiophore stalk length and reduced radial growth. The conidiophore stalk length and radial growth of *A. niger* strain N400 were determined and compared to N401 and N402. The length of N400 conidiophore stalks (2.52 ± 0.40 mm) was reduced in N401 and N402 to 0.66 ± 0.14 mm and 0.34 ± 0.06 mm, respectively. Whereas N400 reached a colony diameter of 6.7 ± 0.2 cm after 7 days, N401 and N402 displayed reduced radial growth phenotype (4.3 ± 0.1 and 4.1 ± 0.1, respectively). To identify the mutations (dubbed *cspA* and *cspB*) responsible for the phenotypes of N401 and N402, the genomes were sequenced and compared to the N400 genome sequence. A parasexual cross was performed between N400 and N402 derivatives to isolate segregants which allowed cosegregation analysis of single nucleotide polymorphisms and insertions and deletions among the segregants. The shorter conidiophore stalk and reduced radial growth in N401 (*cspA*) was found to be caused by a 9-kb deletion on chromosome III and was further narrowed down to a truncation of NRRL3_03857 which encodes a kinesin-like protein homologous to the *A. nidulans* UncA protein. The mutation responsible for the further shortening of conidiophore stalks in N402 (*cspB*) was found to be caused by a missense mutation on chromosome V in a hitherto unstudied C2H2 transcription factor encoded by the gene NRRL3_06646. The importance of these two genes in relation to conidiophore stalk length and radial growth was confirmed by single and double gene deletion studies. The mutations in the laboratory strain N402 should be taken into consideration when studying phenotypes in the N402 background.

## Introduction

Conidiophores are reproductive structures that enable filamentous fungi to produce and disseminate spores. These asexually derived spores are called conidia. Like in other Aspergilli, the process of conidiation or conidiophore formation in *Aspergillus niger* starts with the transformation of a hyphal cell into a characteristic foot cell from which the conidiophore will emerge (Tanaka and Yanagita, 1963). Conidiophore formation continues with the formation of an aerial hyphae of about 6–7 μm in diameter which is called the conidiophore stalk (Adams et al., 1998; Krijgsheld et al., 2013). The length of the conidiophore stalk of the *A. niger* strain IMI41873 has been reported to be around 1,000 μm (Anderson and Smith, 1971). However, variation in conidiophore stalk length occurs among different natural isolates of *A. niger* (Seekles and Ram, unpublished data). Conidiophore stalks of *Aspergillus nidulans* are substantially shorter compared to *A. niger* and extend about 100 μm into the air (Adams et al., 1998). When the stalk reaches its maximum length, the tip of the stalk swells and forms a vesicle (Adams et al., 1998; Krijgsheld et al., 2013). In biserate species like *A. nidulans* and *A. niger*, the vesicle surface buds twice resulting in two layers of sterigmata, which are named metulae and phialides respectively. In uniserate species like *Aspergillus fumigatus*, phialides are directly formed on the vesicle. The phialides give rise to chains of mainly uninucleate conidia and a single conidiophore can produce over 10,000 conidia (Smith et al., 1977; Krijgsheld et al., 2013).

*Aspergillus niger* is an important microorganism for the (recombinant) production of organic compounds, such as citric acid, gluconic acid, and proteins (Pel et al., 2007; Meyer et al., 2011; Li et al., 2020). Commercial citric acid production started over a century ago, and still most of the citric acid used in industry is produced by *A. niger* (Show et al., 2015; Cairns et al., 2018). Besides its use for citric acid production, *A. niger* was also recognized as an efficient producer of extracellular enzymes. Together with the acquired knowledge of cultivating *A. niger* in large bioreactors for citric acid production, this stimulated the research to develop *A. niger* as a host for protein production (Pel et al., 2007). Since the late 1970s, the advent of recombinant DNA techniques facilitated molecular genetics approaches in developing *A. niger* as a cell factory. The long conidiophore stalk of *A. niger* was considered undesirable both in the commercial application of *A. niger* strain CBS120.49 for gluconic acid production and in laboratory practice because it exacerbated cross contamination (Bos et al., 1988; Swart et al., 2001). Therefore, Bos and co-workers isolated a morphological mutant from the gluconic acid producing *A. niger* wild-type strain CBS120.49 (also known as N400, NRRL3 or ATCC9092). The morphological mutant was generated in two independent mutagenesis steps and resulted in the laboratory strain N402 (also known as ATCC64974 or FGSCA733) [Laothanachareon et al., 2018; F. Debets (Wageningen University), personal communication]. The intermediate strain N401 has not been described in detail, but has been preserved and is included in our study. The N402 strain and its auxotrophic and *ku70*^–^ derivatives have been used in numerous molecular genetic studies.

In this study, we aimed to identify the mutation(s) responsible for the reduced conidiophore stalk length of N402. In the course of our studies, we also noticed that N402 displayed a significantly reduced radial growth phenotype compared to N400. We combined genome sequencing of N400, N401, and N402, parasexual crossings between N400 and N402 and cosegregation analysis to identify the mutations responsible for the reduced conidiophore stalk length and the reduced radial growth. Since *A. niger* lacks a known sexual cycle, the parasexual cycle can be used to cross strains to form a diploid from two haploid strains and to subsequently haploidize the diploid strain to obtain haploid segregants (Pontecorvo et al., 1953). The parasexual cycle in *A. niger* has been used previously to obtain segregants for either bulk segregant analysis (Niu et al., 2015; Jørgensen et al., 2020) or co-segregation analysis (van Leeuwe et al., 2020) to identify mutations in genes responsible for the phenotype of particular mutants. We show that deletion of NRRL3_03857 (An15g03580) results in the N401 phenotype, and that this gene is responsible for the reduced radial growth phenotype and responsible for the reduced conidiophore stalk length. Additional deletion of NRRL3_06646 (An16g08800) is responsible for the slightly shorter conidiophore stalks observed in the N402 strain compared to the N401 strain. NRRL3_03857 encodes a kinesin-like protein, homologous to the UncA protein of *A. nidulans* (Zekert and Fischer, 2009) and NRRL3_06646 encodes an uncharacterized C2H2 transcription factor.

## Materials and Methods

### Strains, Media and Growth Conditions

The *A. niger* strains used in this study are listed in Table 1. Strains were grown on liquid or solidified [containing 1.5% (w/v) Scharlau agar] minimal medium (MM) or on complete medium (CM) as described (Arentshorst et al., 2012). Transformants of *A. niger* were isolated and purified as described (Arentshorst et al., 2012) using a final concentration of 100 μg/mL hygromycin or 20 μg/mL phleomycin. Radial growth of strains was assayed by point inoculation of 5 μL filtered spore suspension (1 × 10^6^ spores/mL) in the center of a MM medium containing plate and incubation of the plates for 7 days at 30°C. Uridine and nicotinamide auxotrophies were determined by inoculating strains on MM with or without 10 mM uridine and/or 2.5 μg/mL nicotinamide. *Escherichia coli* DH5α was used for plasmid propagation and cultured at 37°C in Lysogeny broth (LB) medium, with ampicillin (100 μg/mL).

**TABLE 1:** Strains used in this study.

Strain name	Genotype/uridine autotroph or auxotroph	Remark	References
N400	wild-type	NRRL3; ATCC9092; CBS120.49	Bos et al., 1988
N401*	*cspA* in N400		F. Debets (personal communication)
N402*	*cspA, cspB* in N401	ATCC64974	Bos et al., 1988
MA612.27	*kusA: DR-amdS-DR in N400*		This study
MA340.2	*ΔfwnA:hygB, pyrE*^–^ *in* N400, *uridine auxotroph*		This study
JN6.2*	*cspA, cspB, ΔolvA:pyrG*, *ΔnicB:hygB, uridine autotroph*	Derived from N402	Niu et al., 2016
EA14.3	*cspA*^+^*/cspA*^–^, no *cspB*^+^*/cspB*^–^, *fwnA*^+^*/ΔfwnA:hygB, olvA*^+^*/ΔolvA:pyrG*, *pyrE*^+^*/pyrE*^–^, *nicB*^+^*/ΔnicB:hygB, uridine autotroph*	Diploid of MA340.2 and JN6.2	This study
EA14.3#S3	*ΔolvA:pyrG, uridine autotroph*	Segregant of EA14.3	This study
EA14.3#S6	*ΔolvA:pyrG, uridine auxotroph*	Segregant of EA14.3	This study
EA14.3#S8	*ΔfwnA:hygB, uridine autotroph*	Segregant of EA14.3	This study
EA14.3#S4*	*cspB, ΔolvA:pyrG, ΔnicB:hygB, uridine autotroph*	Segregant of EA14.3	This study
EA14.3#S21*	*cspB, ΔolvA:pyrG, uridine autotroph*	Segregant of EA14.3	This study
EA14.3#S28*	*cspB, ΔfwnA:hygB, uridine autotroph*	Segregant of EA14.3	This study
EA14.3#S223*	*cspA, ΔfwnA:hygB, ΔnicB:hygB, uridine autotroph*	Segregant of EA14.3	This study
EA14.3#S224*	*cspA, ΔfwnA:hygB, nicB*^+^, *uridine auxotroph*	Segregant of EA14.3	This study
EA14.3#S91*	*cspA, cspB, ΔolvA:pyrG, uridine auxotroph*	Segregant of EA14.3	This study
BY1.1 (N400 Δ9-kb)	Δ9-kb in MA612.27		This study
BY2.1 (N400 Δ03857)	Δ*NRRL3_03857:hygB in* MA612.27		This study
MA608.2 (N400 Δ06646)	Δ*NRRL3_06646:hygB in* N400		This study
BY4.1 (N400 Δ03857 Δ06646)	Δ*NRRL3_03857:hygB*,Δ*NRRL3_06646:phl in* MA612.27		This study

*cspA and *cspB* in these mutants correspond to the 9-kb deletion on chromosome III and the SNP in NRRL3_06646 on chromosome V, respectively.

Analyses of radial growth and spore density of N400, N401, and N402 were done by point inoculating 5 μl filtered spore suspension (10,000 spores/5 μl) of *A. niger* strains on plates containing MM with glucose. Strains were grown for 7 days at 30°C before measuring the colony diameter and harvesting and counting the spores. Spores were harvested adding 10 ml of 0.9% saline to the plates and were scraped off by a cotton stick. This was repeated once to get the vast majority of the spores from the plate. Spores were collected and filtered over a miracloth filter and the volume was adjusted to 50 ml with 0.9% saline before measuring the spore number using Biorad TC10 cell counter. This experiment was performed in at least four replicates.

### Parasexual Crossing

Formation of heterokaryons and diploid selection was performed as described previously (Arentshorst and Ram, 2018). Strains MA340.2 and JN6.2 were crossed to obtain the diploid strain EA14.3. Haploidization of diploid EA14.3 was performed on CM containing 0.4 μg/mL benomyl and supplemented with 10 mM uridine and 2.5 μg/mL nicotinamide. Haploidization of the diploid strain was observed visually by the formation of fawn and olive-colored segregants. Segregants were randomly picked and single streaked twice on MM with uridine and nicotinamide prior to phenotypic characterization of segregants.

### Conidiophore Stalk Length Measurements

Conidiophore stalk length measurements were performed by cutting out small rectangular agar pieces of the periphery of 7-days old plate cultures containing mycelium and conidiophores. The agar slides were carefully orientated sideways to take images using a Leica EZ4D stereomicroscope with the Leica Application Suite (LAS EZ) software using identical settings (20× magnification rate, 160 exposure and six gain). The length of conidiophore stalks that were clearly visible between the origin of mycelium and the conidial head were measured manually from these pictures using the ImageJ software. For the analysis of the 221 haploid segregants, three stalks were measured and the average of those stalk length measurements was used for further statistical analysis. More detailed growth analysis of parental strain, gene knock-out mutants and selected segregants was done as described above, but an increased number of conidiophore stalks was measured (*n* > 10). Colony diameter was measured after growing point inoculated spores on MM containing glucose for 7 days. Differences in means of conidiophore stalk length or colony diameter were analyzed with ANOVA and subsequent *post hoc* Tukey Honest Significant Difference test. Adjusted *P*-values < 0.001 were considered statistically significant.

### Construction of Gene Deletion/Disruption Mutants

The *ku70/kusA* gene in N400 was disrupted by PCR amplification of the curable *kusA* deletion construct containing the *amdS* selection marker (Carvalho et al., 2010) using primers ku70P1NotI and ku70P4KpnI (Supplementary Table 1). The 4,815-bp long PCR product was introduced directly to N400 and transformants were selected based on the *amdS* selection marker. Transformants were purified and genomic DNA was isolated for Southern blot analysis as described (Arentshorst et al., 2012). Genomic DNA was digested with *Nco*I and probed with a part of the *ku70 gene* (Supplementary Figure 1). Proper deletion was confirmed in MA612.27 and this strain was used for further studies.

MA340.2 was obtained by deleting the *fwnA/pksA* gene in N400 using a split marker approach (Arentshorst et al., 2012) with the primers listed in Supplementary Table 1. Split marker fragments *pksA-hygR 5*′ (3329-bp) and *pksA-hygR 3*′ (3012-bp) were introduced to N400 and a fawn-colored transformant was obtained and purified. This mutant was grown on 5′Fluoro-orotic uracil to select for a *pyrG*^–^ or *pyrE*^–^ mutant as described (Arentshorst et al., 2012). A uridine requiring mutant (MA340.19) was obtained and purified, and by transformation of either the *pyrG* gene or the *pyrE* gene it was found that the mutant had a mutation in *pyrE* as only the *pyrE* containing plasmid complemented the uridine auxotrophy (data not shown).

Strain BY1.1 in which the 9-kb deletion on chromosome III was constructed in N400 was made using CRISPR-Cas9 mediated genome editing as described by van Leeuwe et al., 2019. The CHOPCHOP algorithm (Labun et al., 2019) was used to select a specific PAM site in the 9-kb region and primers were designed to clone these sgRNA in pFC332. The primers for constructing the sgRNA are listed in Supplementary Table 1. The final plasmid containing sgRNA1 (pBY1.1) was verified by sequencing. For constructing the donor DNA, primers 5′FlankFw and 5′flankRv were used to amplify the 5′ flank and primers 3′FlankFw and 3′FlankRv were used to amplify the 3′ flank of the 9-kb region. 3′FlankFw and 5′FlankRv have complementary tails that allows fusion PCR. After PCR amplification of the single flanks, fragments were purified from gel and fused with PCR using primers 5′FlankFw and 3′FlankRv, creating the donor DNA construct. Plasmids pBY1.1 along with the donor DNA was transformed to MA612.27. Diagnostic PCR was performed on the genomic DNA and strain BY1.1 was selected for further analysis (Supplementary Figure 2). Primers used for diagnostic PCR can be found in Supplementary Table 1.

To create strain BY2.1, NRRL3_03857 was replaced with hygromycin resistance cassette in MA612.27 using split marker fragments. Construction of the split marker fragments was done as using primers indicated in Supplementary Table 1. Transformants were checked for proper deletion with diagnostic PCR (Supplementary Figure 3).

MA608.2 (N400 Δ06646) from N400 was also made using the split marker approach using hygromycin resistance as selection marker. Split marker fragments were PCR amplified as described previously (Arentshorst et al., 2015) using primers indicated in Supplementary Table 1. *ΔNRRL3_06646*:*hygB* split marker fragments were transformed to strain N400 and transformants were purified twice on selective media. Genomic DNA of eight transformants was isolated for Southern blot analysis as described (Arentshorst et al., 2012), digested with *Bam*HI and probed with the 3′ flank of NRRL3_06646 (Supplementary Figure 4). Deletion of NRRL3_06646 was confirmed in strain MA608.2 and this strain was used further.

To make the double deletion mutant of NRRL3_06646 and NRRL3_03857 (BY4.1), the NRRL3_06646 gene was replaced with the phleomycin selection marker in BY2.1 (N400 Δ03857). Again, split marker fragments were generated using the primers listed in Supplementary Table 1. Diagnostic PCR to confirm deletion of both NRRL3_06646 and NRRL3_03857 is show in Supplementary Figure 5.

### Genome Sequencing of N400, N401, and N402

Genomic DNA of *A. niger* strains was isolated as described (Arentshorst et al., 2012). The genomic DNA was further column-purified (NucleoSpin Plant II, Macherey-Nagel) for whole genome sequencing. The genomes of were sequenced at the Genome Quebec Innovation (QC, Canada) using the Illumina HiSeqX platform to about 50-fold coverage. Mapping of DNA reads to the NRRL3 genome was done using Bowtie2 (Langmead and Salzberg, 2012). Sequence differences [single nucleotide polymorphisms (SNPs) and insertions and deletions (indels)] were detected with Pilon (Walker et al., 2014). Indels were verified by visualizing read mappings in the Integrative Genome Viewer (Robinson et al., 2017). The Sequence Read Archive accession number for the N400, N401, and N402 read sets is PRJNA693511.

## Results

### Analyses of Conidiophore Stalk Length and Radial Growth of N400, N401, and N402

**FIGURE 1:**
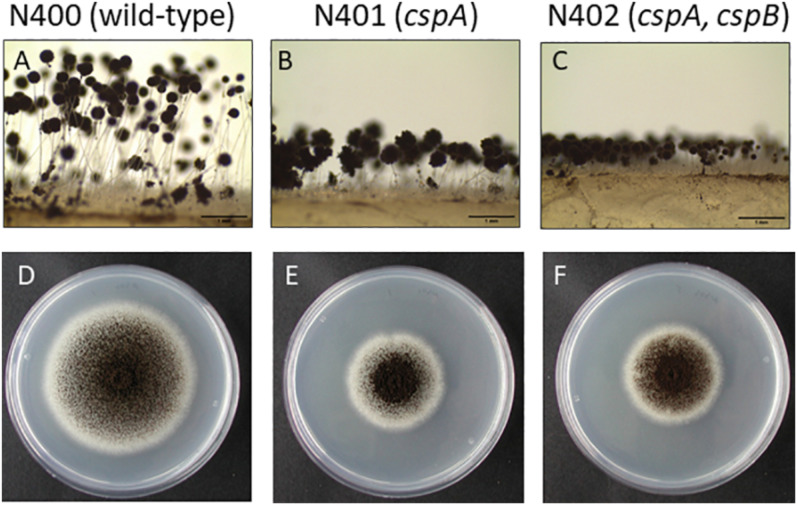
Phenotypic analysis of *Aspergillus niger* N400, N401, and N402. **(A–C)** Conidiophore stalk pictures of N400 **(A)**, N401 **(B)**, and N402 **(C)**. Proposed mutations in N401 (*cspA*) and N402 (*cspA* and *cspB*) are indicated. Bars represent 1 mm. **(D–F)** Radial growth pictures of N400 **(D)**, N401 **(E)**, and N402 **(F)**. Spores were point inoculated on MM containing glucose and grown for 5 days before taking the pictures.

**FIGURE 2:**
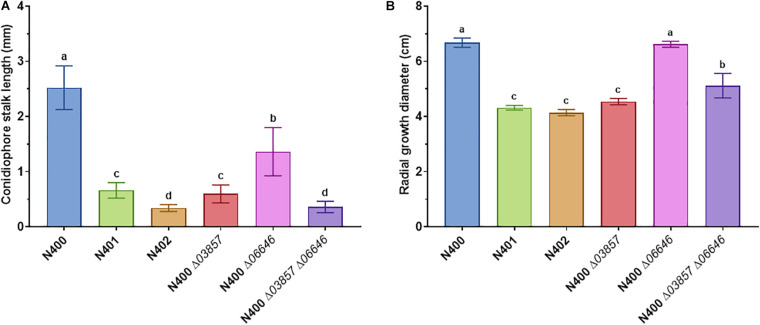
Statistical analysis of conidiophore stalk length and radial growth diameter of selected *A. niger* strains. **(A)** Conidiophore stalk length measurements (*n* > 10) and **(B)** radial growth measurements (*n* = 3) of N400, N401, and N402, and mutants with targeted deletion of *cspA* (*ΔNRRL3_03857*), *cspB* (*ΔNRRL3_06646*), and *cspA* and *cspB* (*ΔNRRL3_03857 ΔNRRL3_06646*). The means represented with different letters are significantly different (Tukey’s HSD, *P*-value < 0.001).

Strains N401 and N402 are derivatives of wild-type *A. niger* strain N400 and were obtained by two consecutive rounds of UV mutagenesis [Laothanachareon et al., 2018; F. Debets (Wageningen University), personal communication]. Stalk lengths of N400, N401, and N402 were determined as described in the “Materials and Methods” section. The average length of the conidiophore stalks was 2.52 ± 0.40 mm in the wild-type strain N400, 0.66 ± 0.14 mm in N401, and 0.34 ± 0.06 mm in N402 (Figures 1A–C and Table 2). Based on the means and variations observed, these differences in conidiophore stalk length between the three strains were found to be statistically significant (Figure 2 and Supplementary Table 2). In addition to the shorter conidiophore stalks, both the N401 and the N402 strains showed a reduced radial growth phenotype compared to N400 (Figures 1D,E, 2 and Table 2). It also has been reported that the spore densities differ between the three strains (Parenicova, 2000). Therefore the conidiation efficiency of N400, N401, and N402 was determined by isolating spores from a colony that was grown for 7 days on MM containing 50 mM glucose. As shown in Supplementary Figure 6, the density of spores (spores/mm^2^) did not differ significantly between the strains. We hypothesized that the phenotypes observed in N401 are caused by a mutation (called *cspA*) resulting in reduced radial growth and reduced conidiophore stalk length and that N402 harbors an additional mutation (*cspB*) that causes a further reduction in conidiophore stalk length. Accordingly, we hypothesized that the reduced radial growth of N401 and N402 is caused by the *cspA* mutation, and since the radial growth of N402 is not further reduced compared to that of N401, *cspB* is not affecting the radial growth.

**TABLE 2:** Phenotypic properties of relevant strains derived from N400.

Strain name	*csp* related phenotype	Conidiophore stalk length (mm)	Colony diameter (cm)	9 kb region present/absent*^1^	SNPs in NRRL3_06646*^2^	Group	Trivial name
N400	–	2.52 ± 0.40	6.68 ± 0.17	present	TTC-CAA (wt)	Group I*^3^	
N401	*cspA*	0.66 ± 0.14	4.32 ± 0.08	absent	TTC-CAA (wt)	Group III*^3^	
N402	*cspA, cspB*	0.34 ± 0.06	4.14 ± 0.11	absent	TTT-TAA (mutant)	Group IV*^3^	
MA340.2	*–*	2.02 ± 0.53	6.8	n.d.*^4^	n.d.	Group I	
JN6.2	*cspA, cspB*	0.33 ± 0.01	4.0	absent	n.d.	Group IV	
EA14.3	–	1.78 ± 0.35	7.0	n.d.	n.d.	Group I	
EA14.3#S3	–	2.11 ± 0.33	6.5	present	TTC-CAA (wt)	Group I	Long
EA14.3#S6	–	2.19 ± 0.40	6.2	present	TTC-CAA (wt)	Group I	Long
EA14.3#S8	–	2.53 ± 0.46	6.6	present	TTC-CAA (wt)	Group I	Long
EA14.3#S4	*cspB*	1.12 ± 0.20	5.9	present	TTT-TAA (mutant)	Group II	Intermediate
EA14.3#S21	*cspB*	1.58 ± 0.24	6.7	present	TTT-TAA (mutant)	Group II	Intermediate
EA14.3#S28	*cspB*	1.48 ± 0.26	6.5	present	TTT-TAA (mutant)	Group II	Intermediate
EA14.3#S223	*cspA*	0.50 ± 0.06	3.5	absent	TTC-CAA (wt)	Group III	Short
EA14.3#S224	*cspA*	0.58 ± 0.17	3.8	absent	TTC-CAA (wt)	Group III	Short
EA14.3#S91	*cspA, cspB*	0.36 ± 0.03	4.2	absent	TTT-TAA (mutant)	Group IV	Very short
BY1.1 (N400 Δ9kb)	*cspA*	n.d.*^5^	3.9	absent	n.a.*^6^	Group III	
BY2.1 (N400 Δ03857)	*cspA*	0.60 ± 0.16	4.48 ± 0.29	n.d.	n.a.	Group III*^3^	Short
MA608.2 (N400 Δ06646)	*cspB*	1.36 ± 0.44	6.62 ± 0.11	n.d	n.a.	Group II*^3^	Intermediate
BY4.1 (N400 Δ03857 Δ06646)	*cspA, cspB*	0.36 ± 0.10	5.15 ± 0.50	n.d.	n.a.	Group IV*^3^	Very short

*^1^The presence of the 9 kb region was verified *via* diagnostic PCR (Supplementary Figure 4). *^2^SNP was verified by PCR amplification and sequencing of the region harboring the SNP (data not shown). *^3^Supplementary Table 2. *^4^n.d., not determined. *^5^Pictures taken show that the condiophore stalk in the 9-kb deletion strain is similar to N401. *^6^n.a., not applicable.

### Parasexual Crossing Between N400 and N402 to Dissect the *cspA* and *cspB* Mutations

To gain genetic evidence that the reduced radial growth and conidiophore stalk length phenotype of N401 are caused by the same mutation (*cspA*) and that the further reduction in conidiophore stalk length in N402 is caused by an additional mutation (*cspB*), we carried out a crossing between N400 and N402. To select and identify diploids derived from the parasexual genetic cross (Arentshorst and Ram, 2018), auxotrophic and color markers were introduced in the N400 background (MA340.2; *ΔfwnA:hygB, pyrE*^–^) and N402 background (JN6.2; *ΔolvA:pyrG*, *ΔnicB:hygB*). The conidiophore stalk length and radial growth of the N400 and N402-derived auxotrophic mutants were similar to the parental strain (Table 2). The obtained diploid from the cross (EA14.3) showed a similar conidiophore stalk length and radial growth to those of the N400 strain, indicating that the mutation(s) responsible for these phenotypes are recessive (Table 2).

**FIGURE 3:**
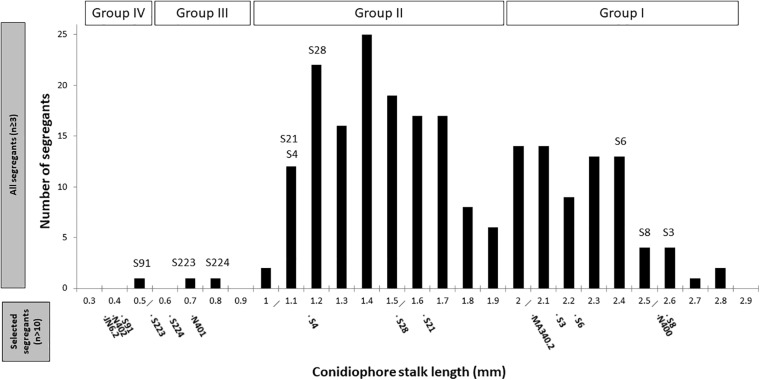
Distribution of conidiophore stalk lengths of 221 segregants. Measurements of the stalk length (*n* ≥ 3) were performed from the peripheral end of the colonies, as explained in the section “Materials and Methods.” The selected representative segregants for further analysis are indicated. Conidiophore stalk length of the control strains and representative segregants measured subsequently in more detail (*n* > 10) are indicated in the bottom part of the x-axis.

Segregants from the diploid were obtained by plating out spores of the diploid strain on benomyl containing CM as described (Arentshorst and Ram, 2018). A total of 221 haploid segregants were purified and analyzed for their conidiophore stalk length and radial growth. The distribution of the conidiophore stalk length of the segregants as well as the N400 derived strains is shown in Figure 3. We identified four groups (Group I to IV) of segregants that were not equally distributed based on their conidiophore stalk lengths. The first type (Group I) consists of segregants with an average stalk length similar to that of N400. Segregants with a stalk length of 1.9 mm and higher are considered to be part of this group. The second type (Group II) consists of segregants with a conidiophore stalk length between 0.9 and 1.9 mm. The third type (Group III) consists of only two segregants (S223 and S224) with a conidiophore stalk length phenotype similar to that of N401. Group IV has a conidiophore stalk length phenotype similar to that of N402 and is represented by a single segregant (S91). Representative segregants from each group were selected (Figure 3) and their radial growth phenotype was analyzed. As shown in Table 2 and Supplementary Figure 7A, segregants with short conidiophores (Group III and IV) were found to have a reduced radial growth phenotype, while mutants with intermediate (Group II) and long (Group I) conidiophores had a radial growth diameter similar to that of N400. In addition, the presence of four conidiophore stalk length groups was supported by one-way ANOVA analysis of the representative strains as shown in Supplementary Figure 7B. This analysis indicated, likely because of the limited number of measurements of the conidiophore stalk length of the segregants (*n* = 3), the presence of more than four and overlapping groups.

These results can be explained by the proposed *cspA* and *cspB* mutations where the *cspA* mutation causes reduced conidiophore stalk length and reduced radial growth. The *cspB* mutation causes further reduction of conidiophore stalk length when combined with *cspA*. By itself and dissected from the *cspA* mutation, the *cspB* mutation is likely to be represented by Group II segregants with a reduced conidiophore stalk length, but without impaired radial growth. The observation that the reduced conidiophore stalk length and reduced radial growth cosegregate in the Group III and Group IV segregants suggests strongly that both phenotypes are caused by a single mutation (*cspA*). Therefore, based on our hypothesis, Group I segregants are not expected to have the *cspA* and *cspB* mutations, while Group II segregants are expected to have the *cspB* mutation, Group III the *cspA* mutation, and Group IV both the *cspA* and *cspB* mutations. Although we expected equal distribution of the mutations and their related phenotypes among the segregants, the mutations present in Group III and IV segregants are clearly underrepresented, indicating that there is a preference to maintain chromosomes with the wild-type *cspA* and *cspB* alleles (see section “Discussion”).

We also examined whether the genetic markers (either the uridine and nicotinamide auxotrophic markers or the color markers) had an effect on the conidiophore stalk length. Supplementary Figure 8 shows that the conidiophore stalk length distribution of the segregants, regardless of their auxotrophic and color phenotypes, is similar to the conidiophore stalk length distribution of all 221 segregants, indicating that the auxotrophic and color genetic markers did not have an effect on the stalk length. Despite the unequal distribution of the segregants, the segregation analysis yielded an interesting set of segregants with distinct phenotypes (Supplementary Figure 7) to perform co-segregation analysis by genome sequencing.

### Genome Sequencing of N400, N401, and N402

To identify candidate SNPs and indels which could be responsible for the phenotypes of N401 and N402, the genomic DNA of N401 and N402 was isolated, sequenced, and compared to the N400 genome sequence. We identified 35 SNPs in coding regions when comparing N400 and N401, and an additional 29 SNPs when comparing N401 and N402 (Supplementary Table 3). In addition to these SNPs, 14 indels were detected in N401 compared to N400 and a single insertion was detected in N402 compared to N401 (Supplementary Table 4). The largest deletion in N401 is a 9,344 bp deletion on chromosome III detected in N401 and N402 compared to N400. Within this 9-kb region, three open reading frames (ORFs) (NRRL3_03857, NRRL3_03858, and NRRL3_03859) are located. As noted in a previous report (Laothanachareon et al., 2018), NRRL3_03857 encodes a kinesin-like protein which could be responsible for the short-stalk conidiophore phenotype of N402. Previous experiments in the Wageningen University have suggested genetic linkage of the short conidiophore stalk phenotype with markers on chromosome III [F. Debets (Wageningen University), personal communication]. Therefore, the involvement of this deletion was further examined.

### Identification of *cspA*

Since the 9-kb deletion is a candidate to be responsible for phenotypes of N401 and N402, a co-segregation analysis was performed on nine selected segregants and the parental strains. As shown in Table 2 and Supplementary Figure 7, in all segregants with a (very) short conidiophore stalk (Group III and IV) the reduced conidiophore stalk length and radial growth phenotype were correlated with the 9-kb deletion. Targeted deletion of the 9-kb region using CRISPR-Cas9 in MA612.27, a *ku70*^–^ derivative of N400 background, further confirmed that this region is required for conidiophore stalk length and radial growth observed in the wild-type strain N400. The length of the conidiophore stalk and the radial growth of the 9-kb deletion mutant [BY1.1 (N400 Δ9-kb)] were similar to those of N401 (data not shown). Of the three predicted ORFs on the 9-kb region, the kinesin-like protein was most likely to be the candidate to be responsible for the phenotype (Laothanachareon et al., 2018). Therefore, the NRRL3_03857 gene was deleted in MA612.27 (Supplementary Figure 3). The phenotypic analysis of the NRRL3_03857 deletion mutant BY2.1 (N400 Δ03857) showed that its conidiophore stalk length and radial growth phenotype was similar to that of N401 (*cspA*) (Figure 2 and Table 2). We conclude that the kinesin-like protein encoded by NRRL3_03857 is responsible for the short conidiophore stalk (observed in N401) and the reduced radial growth.

### Identification of *cspB*

Strain N402 was isolated following UV-mutagenesis of strain N401 because it displayed a shorter conidiophore stalk than N401 (Laothanachareon et al., 2018; F. Debets, Wageningen University, personal communication). Strain N402 has a statistically significantly shorter conidiophore stalk compared to N401 (Figures 1, 2). SNP analysis between N401 and N402 revealed 36 SNPs in the coding regions (Supplementary Table 3). Two adjacent transitions (both C to T) were found in the coding region of NRRL3_06646. NRRL3_06646 encodes an 861 amino acid long C2H2 Zn finger transcription factor. The first SNP does not affect the amino acid sequence at position 56 (TTC to TTT, phenylalanine), but the second SNP (CAA to TAA) introduces a stop codon. Of the 36 SNPs found between N401 and N402, the second SNP in NRRL3_06646 was the only SNP that introduced a stop codon. To examine whether the stop codon in NRRL3_06646 is responsible for the *cspB* phenotype, an SNP co-segregation analysis on the haploid segregants from the cross between N400 and N402 strains was performed. Among the segregants, the mutation was found in the single Group IV segregant with the very short conidiophore stalk phenotype (S91) as well as the three Group II segregants with the intermediate conidiophore stalk phenotype (Table 2). The cosegregation is consistent with the idea that the mutation in NRRL3_06646 caused reduced conidiophore stalk length of N402 compared to that of N401 and with an intermediate conidiophore stalks of the Group II segregants. To confirm the role of NRRL3_06646, the gene was deleted in N400 (Supplementary Figure 4). Deletion of NRRL3_06646 in N400 results in a reduced conidiophore stalk length, similar to that of the Group II segregants (Figure 2A and Table 2). Deletion of NRRL3_06646 did not lead to a reduction in radial growth (Figure 2B and Table 2). These results confirm that the *cspB* phenotype is caused by inactivation of NRRL3_06646.

The identification of the *cspA* (NRRL3_03857) and *cspB* (NRRL3_06646) genes also allowed the possibility to combine deletion of these genes to examine whether this would result in the phenotype observed in N402. Therefore, the ΔNRRL3_03857 ΔNRRL3_06646 double deletion mutant was constructed and verified by diagnostic PCR (Supplementary Figure 5). Phenotypic analysis of the double deletion mutant showed a conidiophore stalk length comparable to that N402 (Figure 2A and Table 2), indicating that both the kinesin-like protein (CspA) and the C2H2 transcription factor (CspB) are required for conidiophore stalk development in *A. niger*.

## Discussion

For many applied and fundamental research studies in *A. niger*, derivatives of the NRRL3/N400 wild-type isolate have been used. *A. niger* strain CBS120.49 (N400) was selected for the commercial production of gluconic acid in the 1980s. To prevent cross contaminations of *A. niger* spores short conidiophore stalk mutants were isolated that were later on also used to make auxotrophic strains for crossing experiments and gene transformations (Bos et al., 1988; Swart et al., 2001). The most used auxotrophic strains are uracil deficient strains (called *pyrA* and *pyrG* in *A. niger*, which are the equivalent of the *Saccharomyces cerevisiae ura3* marker). Two laboratories generated this mutant independently of each other. The uridine requiring mutant (named *pyrA)* made in the Wageningen laboratory is named N593 (Goosen et al., 1987) and the uridine requiring mutant (named *pyrG*) isolated in the group of van den Hondel is called AB4.1 (van Hartingsveldt et al., 1987). Both uridine-requiring mutants and *Δku70* derivatives of both strains are used in *A. niger* research laboratories.

Until now, the phenotypes of the N401 and N402 strains have not been carefully described. The detailed measurements of conidiophore stalk length and radial growth conducted in this study showed that N402 has a significantly reduced conidiophore stalk length and colony diameter compared to N400. To sort out the molecular basis for the short conidiophore stalk and reduced radial growth, the genomes of N400 (NRRL3), N401 and N402 were sequenced and compared with each other. SNP analysis identified 35 SNPs between N400 and N401 and 29 SNPs between N401 and N402 located in 34 and 23 predicted ORFs, respectively. To perform the co-segregation analysis, segregants of a diploid strain obtained from a parasexual cross between a N400 derivative (MA340.2, *ΔfwnA:hygB, pyrE*^–^) and a N402 derivative (JN6.2, *ΔolvA:pyrG, ΔnicB:hygB*) were obtained and analyzed. Both the color markers and *nicB* auxotrophic marker were more or less equally distributed among the 221 segregants (121 fawn and 100 olive; 132 nicotinamide autotrophs and 89 nicotinamide auxotrophs). Please note that the segregation of the *pyrE* or *pyrG* markers is more difficult to interpret because of the *pyrG* mutation at the endogenous *pyrG* locus in JN6.2. Therefore, the uridine-requirement phenotype cannot be concluded to be caused by mutations in the endogenous *pyrG* and/or *pyrE* loci. Of the 221 segregants, 59 were uridine requiring and 162 were prototrophic for uracil. The conidiophore stalk length distribution of the segregants was analyzed. If a single mutation was responsible for the short conidiophore stalk phenotype, two populations would be observed with one having a stalk length similar to that of N400 and the other having a stalk length similar to that of N402. As shown in Figure 3, such a distribution was not found. Instead, we found a distribution that can be better explained by two mutations (*cspA* and *cspB*) responsible for the short conidiophore stalk phenotype that are unequally represented in the segregants. Two large size populations were found with an average conidiophore stalk length of 2.21 mm (similar to that of N400) (74 segregants) and 1.39 mm (144 segregants). We showed that the reduction in conidiophore stalk length is caused by mutation in NRRL3_06646 (CspB) and the *cspB* mutation is distributed more or less equally among the segregants. The other postulated mutation, *cspA*, is not distributed equally as only three segregants were found to have this mutation. Two segregants were found to have only the *cspA* mutation (S223 and S224) and a single segregant (S93) to have both mutations (*cspA* and *cspB*). The uneven distribution of the *cspA* mutation in the segregants suggests that there is a strong bias to maintain the *cspA* wild-type locus during the segregation process. Segregation of the diploid into haploid cells is accomplished by adding the microtubule disturbing agent benomyl to the growth medium. As discussed below in more detail, the *cspA* related phenotype is caused by the inactivation of a kinesin-like motor protein which is a microtubule dependent motor protein. Possibly, the mutant lacking this protein is more sensitive to benomyl, causing such segregants to have a growth defect on benomyl containing medium and the observed bias.

The short conidiophore stalk and reduced radial growth observed on strain N401 (*cspA* phenotype) is caused by the loss of function of gene NRRL3_03857. In N401, this gene is partially deleted because of the 9,344-bp long deletion on chromosome III. This 9-kb region contains three predicted genes, encoded by NRRL3_03857, NRRL3_03858, and NRRL3_03859. NRRL3_03858 and NRRL3_03859 are predicted to encode hypothetical proteins of 170 and 150 amino acids long, respectively. The 9-kb deletion results in deletion of the first 1,399 bp of the 5,008-bp long ORF of NRRL3_3857, thereby deletion of the first 438 amino acids in the N-terminal part of the protein. We deleted NRRL3_3857 in N400 and confirmed that ΔNRRL3_3857 yields the *cspA* phenotype in N401. CspA protein encoded by NRRL3_3857 displays 84.5% amino acid sequence identity with the UncA protein of *A. nidulans*, and therefore UncA is considered to be the functional homolog of CspA. Kinesins are molecular motor proteins that can move along microtubules, and 17 protein families have been defined based on the sequence similarities of their motor protein domain (Lawrence et al., 2004; Wickstead and Gull, 2006). UncA encodes a kinesin-3 member within the superfamily of kinesins. This plus-end directed motor protein harbors its motor domain at the N-terminus. Other domains include a pleckstrin homology (PH) domain for the binding of membranous cargo and a forkhead-associated (FFH) domain (Klopfenstein et al., 2002). The UncA homolog of *A. niger*, CspA, has the same protein domains conserved. Deletion of the *uncA* gene in *A. nidulans* resulted in strain with a reduced growth rate and a seemingly more compact phenotype and increased branching (Zekert and Fischer, 2009). The organization of the microtubules and distribution of nuclei or mitochondria was not affected by *uncA* deletion. Studies in *A. nidulans* showed that UncA transports vesicles preferentially along a detyrosinated type of microtubule which is stable during mitosis and therefore secures transport of secretion related vesicles continuously even during mitosis (Riquelme et al., 2003; Zekert and Fischer, 2009).

The second mutation identified in N402 which contributes to the short conidiophore stalk phenotype is a loss-of-function mutation in an uncharacterized C2H2 transcription factor (NRRL3_06646). The mutation introduces a stop codon after amino acid 56 of the 861 amino acid long protein. Homology searches indicated that the transcription factor is conserved, but the function is unknown. The function of this transcription factor in relation to conidiophore development as well as the genes that are likely under the control of this transcription factor should be studied in more detail.

An important consideration is to which extent the mutations in the kinesin-like protein and the transcription factor that lead to shorter conidiophore stalk and reduced radial growth identified in this study might affect the conclusions reached in previous studies. Schäfer et al. (2020) performed a comparative study in which different *A. niger* strains were compared for growth under controlled conditions. This analysis also included a comparison between N400 and N402. The results indicated that the growth profile in bioreactor was similar, although the N402 strain reached a higher maximum dry biomass level compared to N400 (2.28 ± 0.12 g/L for N400 vs 3.38 ± 0.07 g/L for N402) (Schäfer et al., 2020). The morphology of the mycelium of the N400 and N402 strains during cultivation on pectin was also examined and compared. In comparison to N400 which displays a rather dispersed growth behavior, the N402 showed a strong pellet-like growth. Toward the end of the cultivation the N400 strain also displayed a more pelleted growth behavior. The protein production capacity of both strains was also assessed, and importantly, the protein concentrations in the medium are highly comparable peaking at 157 ± 10 mg/L (90h) for N400 and 162 ± 5 mg/L (81 h) for N402. The total pectinase and endo-specific polygalacturonase activity of medium samples was also analyzed. Compared to N400, the N402 strain had both reduced total pectinase and endo-polygalacturonase activity (Schäfer et al., 2020). A possible role for the *cspA* or *cspB* mutations in regulating pectinase expression was also suggested by the reduced expression of *plyA* [pectate lyase A and *pelA* (pectin lyase A)] in N400 compared to N402 derivatives (Parenicova, 2000). These comparative studies indicate that there are no major differences between N400 and N402 in terms of protein production in general, but that the production of some endo-polygalacturonases or lyases might be affected in the N402 strain. Expression of endo- polygalacturonases in *A. niger* N402 is in general very low, even under inducing conditions (Alazi et al., 2016). Whether the expression of some endo-polygalacturonases is altered in N400 requires further investigation.

The N402 strain is a laboratory strain for numerous studies. Those studies in which mechanisms related to secretion or polarized growth (e.g., Kwon et al., 2010, 2013, 2014; Krijgsheld et al., 2012; Fiedler et al., 2018) have been studied using the N402 strain require some attention and possible re-evaluation given the new findings that strain N402 is defective in a gene encoding a kinesin-like protein. Although the overall effect of the protein secretion capacity of N402 seems not to be affected, one has to keep in mind the localization and dynamics of the secretory system components, such as secretory vesicles, and secretion might be influenced by the lack of the kinesin-like 3 motor proteins. Studies using *A. niger* as a model investigating transcriptional regulation of (biopolymer degrading) enzyme production are also numerous (e.g., van Peij et al., 1998; Yuan et al., 2008; de Souza et al., 2011; Delmas et al., 2012; Kowalczyk et al., 2015; Alazi et al., 2016). Based on the observation that the expression of pectinases might be altered in the N402 strain (Parenicova, 2000; Schäfer et al., 2020), mutations in N402 might also have an effect on the expression of these enzymes network. To circumvent possible effects of the mutations introduced in N402, N400 should be considered as a true wild-type strain and used preferably. To facilitate genome editing in N400 a *ku70*^–^ *(kusA*^–^) derivative of N400 (MA612.27) is available and together with CRISPR-Cas9-based genome editing systems (van Leeuwe et al., 2019) efficient and rapid genome editing in strain N400 is feasible.

## Data Availability Statement

The datasets presented in this study can be found in online repositories. The names of the repository/repositories and accession number(s) can be found at: https://www.ncbi.nlm.nih.gov/sra, PRJNA693511.

## Author Contributions

ED: conceptualization, investigation, validation, data analysis, methodology, original draft writing, and visualization. MA, BY, and AS: investigation and data analysis. IR: genome sequencing and data analysis. JV: conceptualization and data analysis. AT: conceptualization, genome sequencing, data analysis, and funding. AR: conceptualization, data analysis, original draft writing, funding acquisition, and supervision. All authors contributed to the writing and agree to be accountable for the content of the work.

## Conflict of Interest

The authors declare that the research was conducted in the absence of any commercial or financial relationships that could be construed as a potential conflict of interest.

## Supplementary Material

Supplementary Figure 1 Southern blot analysis to verify *kusA:DR-amdS-DR* disruption in N400. **(A)** Schematic representation of *kusA* locus in N400 and in the mutant. DNA fragments expected to hybridize with the probe after digestion of genomic DNA with *Nco*I are indicated. **(B)** Genomic DNA of putative *kusA:DR-amdS-DR* mutants and control strains N402 and MA234.1 (*kusA:DR-amdS-DR*) was analyzed. Strain MA612.27 was selected for further studies as it displayed the expected band of the digested genomic DNA.

Supplementary Figure 2 Diagnostic PCR to confirm the presence/absence of the 9-kb region in parental strains MA340.2 and JN6.2, and in selected segregants. The segrgants analyzed are S3, S6, S8, S4, S21, S28, S223, S224, and S91. **(A)** Expected band size is 11-kb if the region is present and 2-kb if the region is absent. **(B)** Expected band size is 2.6-kb if the region is present and no band is expected if the region is absent.

Supplementary Figure 3 Diagnostic PCR to confirm the deletion of NRRL3_03857 in MA612.27. **(A,C)** Schematic representation of NRRL3_03847 locus before and after deletion and the sizes of the expected PCR fragments in N400 and Δ*NRRL3_03857* using the indicated primers. **(B,D)** Agarose gel pictures of the PCR products. The position of the 1-kb molecular marker is indicated.

Supplementary Figure 4 Southern blot analysis to verify the deletion of NRRL3_06646 in N400. **(A)** Schematic representation of NRRL3_06646 locus in N400 and in the mutant. DNA fragments expected to hybridize with the probe after digestion of genomic DNA with *Bam*HI are indicated. **(B)** Genomic DNA of putative *ΔNRRL3_06646* mutants and control strain N400 was analyzed. Strain MA608.2 was selected for further studies as it displayed the expected band of the digested genomic DNA.

Supplementary Figure 5 Diagnostic PCR to confirm the deletion of NRRL3_06646 and NRRL3_03857 in N400. **(A)** Schematic representation of NRRL3_03857 locus before and after deletion and the sizes of the expected PCR fragments in N400 and Δ*NRRL3_03857* with the indicated primers. **(B)** Schematic representation of NRRL3_06646 locus before and after deletion and the sizes of the expected PCR fragments in N400 and Δ*NRRL3_06646* using the indicated primers. Transformant 3 with the expected sizes of the PCR products was selected for further experiments and named BY4.1.

Supplementary Figure 6 Analyses of radial growth and spore density in N400, N401, and N402. Spores of *A. niger* strains N400, N401, and N402 were point inoculated (10,000 spores/5 μl) and grown for 7 days at 30°C on plates containing MM with glucose before measuring the colony diameter and harvesting and counting the spores. Means represented with different number of stars are significantly different (Student’s *t* test *P*-value < 0.05).

Supplementary Figure 7 Phenotypic analysis of *A. niger* strain N400 and its derivatives (see Table 1 for details regarding the strains). **(A)** Radial growth diameter measurements and **(B)** Conidiophore stalk length measurements of N400, N401, and N402, mutants with targeted deletion of *cspA* (*ΔNRRL3_03857*), *cspB* (*ΔNRRL3_06646*), and *cspA* and *cspB (ΔNRRL3_03857 ΔNRRL3_06646*), and representative segregants. Means represented with different letters are significantly different (Tukey’s HSD, *P*-value < 0.001).

Supplementary Figure 8 Distribution of conidiophore stalk lengths of the segregants having a specific phenotype in comparison to that of all 221 segregants.

Supplementary Table 1 Primers used in this study.

Supplementary Table 2 Results of one-way ANOVA of the conidiophore stalk lengths of different strains used in this study.

Supplementary Table 3 SNPs identified in coding regions in N401 and N402 compared to N400.

Supplementary Table 4 Indels detected between N400, N401, and N402.
