# Supplementary material for: Genetic Characterization of Mutations Related to Conidiophore Stalk Length Development in Aspergillus niger Laboratory Strain N402
Source: Front Genet. 2021 Apr 20;12:666684. doi: 10.3389/fgene.2021.666684 (PMC8093798; doi:10.3389/fgene.2021.666684)
Supplement: Supplementary Figure 1 — Southern blot analysis to verify kusA:DR-amdS-DR disruption in N400. (A) Schematic representation of kusA locus in N400 and in the mutant. DNA fragments expected to hybridize with the probe after digestion of genomic DNA with NcoI are indicated. (B) Genomic DNA of putative kusA:DR-amdS-DR mutants and control strains N402 and MA234.1 (kusA:DR-amdS-DR) was analyzed. Strain MA612.27 was selected for further studies as it displayed the expected band of the digested genomic DNA. [file Data_Sheet_1.DOCX]

Supplemental Figure 1


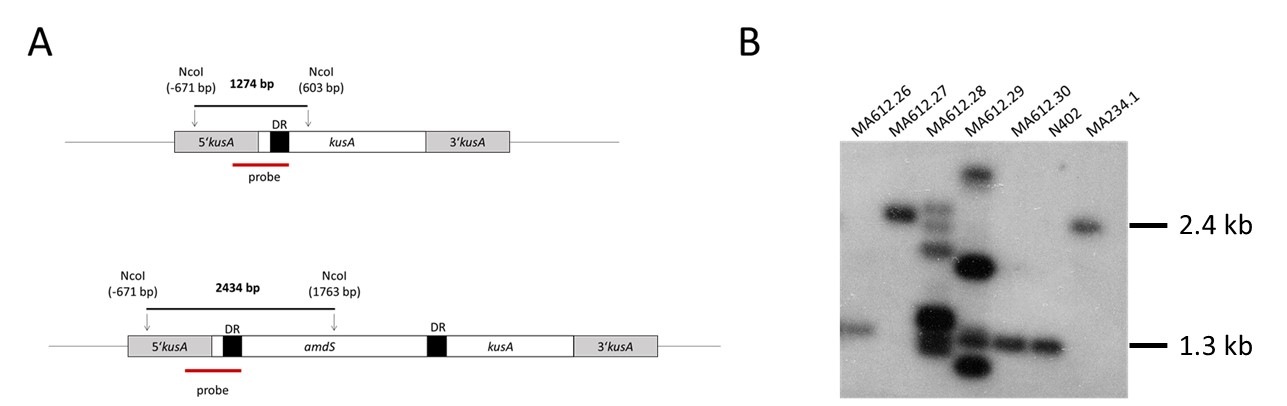


**Supplemental Figure 1**. Southern blot analysis to verify *kusA::DR-amdS-DR* disruption in N400. A) Schematic representation of *kusA* locus in N400 and in the mutant. DNA fragments expected to hybridize with the probe after digestion of genomic DNA with *Nco*I are indicated. B) Genomic DNA of putative *kusA::DR-amdS-DR* mutants and control strains N402 and MA234.1 (*kusA::DR-amdS-DR*) was analyzed. Strain MA612.27 was selected for further studies as it displayed the expected band of the digested genomic DNA.
