# Supplementary material for: Genetic Characterization of Mutations Related to Conidiophore Stalk Length Development in Aspergillus niger Laboratory Strain N402
Source: Front Genet. 2021 Apr 20;12:666684. doi: 10.3389/fgene.2021.666684 (PMC8093798; doi:10.3389/fgene.2021.666684)
Supplement: Supplementary Figure 2 — Diagnostic PCR to confirm the presence/absence of the 9-kb region in parental strains MA340.2 and JN6.2, and in selected segregants. The segrgants analyzed are S3, S6, S8, S4, S21, S28, S223, S224, and S91. (A) Expected band size is 11-kb if the region is present and 2-kb if the region is absent. (B) Expected band size is 2.6-kb if the region is present and no band is expected if the region is absent. [file Data_Sheet_2.DOCX]

Supplemental Figure 2


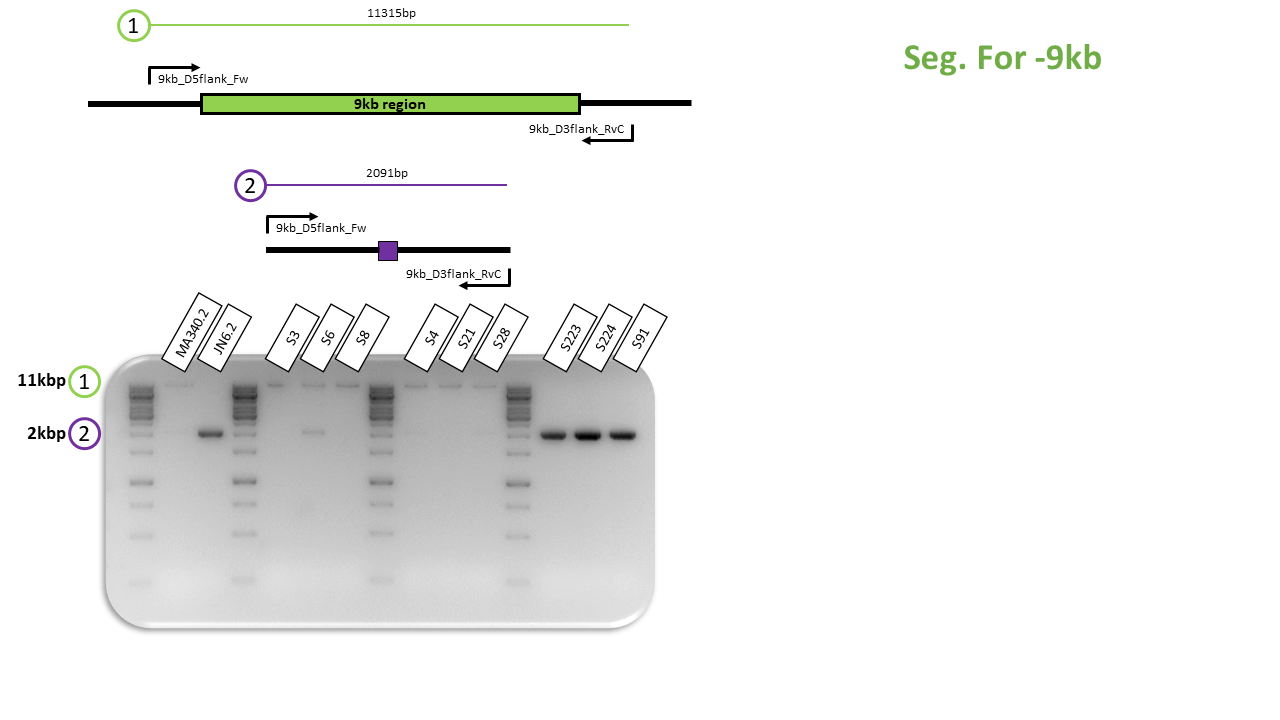


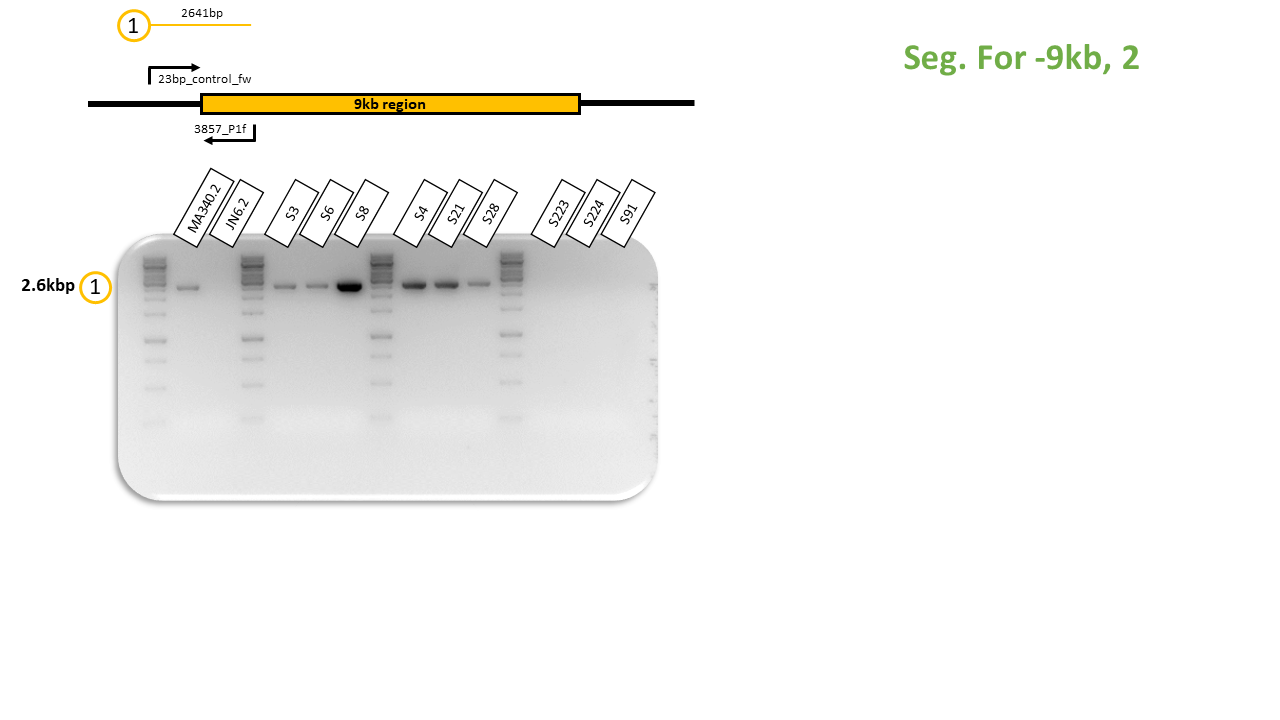


Supplemental Figure 2. Diagnostic PCR to confirm the presence/absence of the 9-kb region in parental strains MA340.2 and JN6.2, and in selected segregants. The segrgants anlysed are S3, S6, S8, S4, S21, S28, S223, S224, and S91. A) Expected band size is 11-kb if the region is present and 2-kb if the region is absent. B) Expected band size is 2.6-kb if the region is present and no band is expected if the region is absent.
