# Supplementary material for: Genetic Characterization of Mutations Related to Conidiophore Stalk Length Development in Aspergillus niger Laboratory Strain N402
Source: Front Genet. 2021 Apr 20;12:666684. doi: 10.3389/fgene.2021.666684 (PMC8093798; doi:10.3389/fgene.2021.666684)
Supplement: Supplementary Figure 3 — Diagnostic PCR to confirm the deletion of NRRL3_03857 in MA612.27. (A,C) Schematic representation of NRRL3_03847 locus before and after deletion and the sizes of the expected PCR fragments in N400 and ΔNRRL3_03857 using the indicated primers. (B,D) Agarose gel pictures of the PCR products. The position of the 1-kb molecular marker is indicated. [file Data_Sheet_3.DOCX]

Supplemental Figure 3

**A**


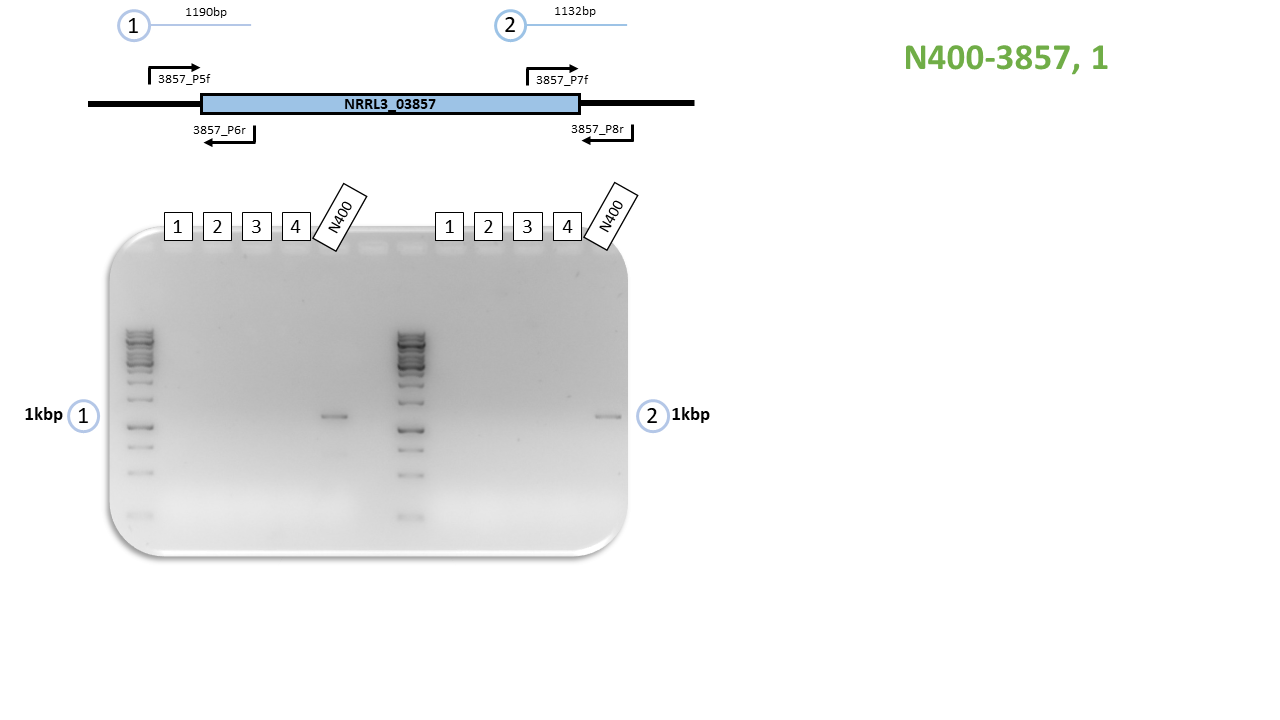


**B**


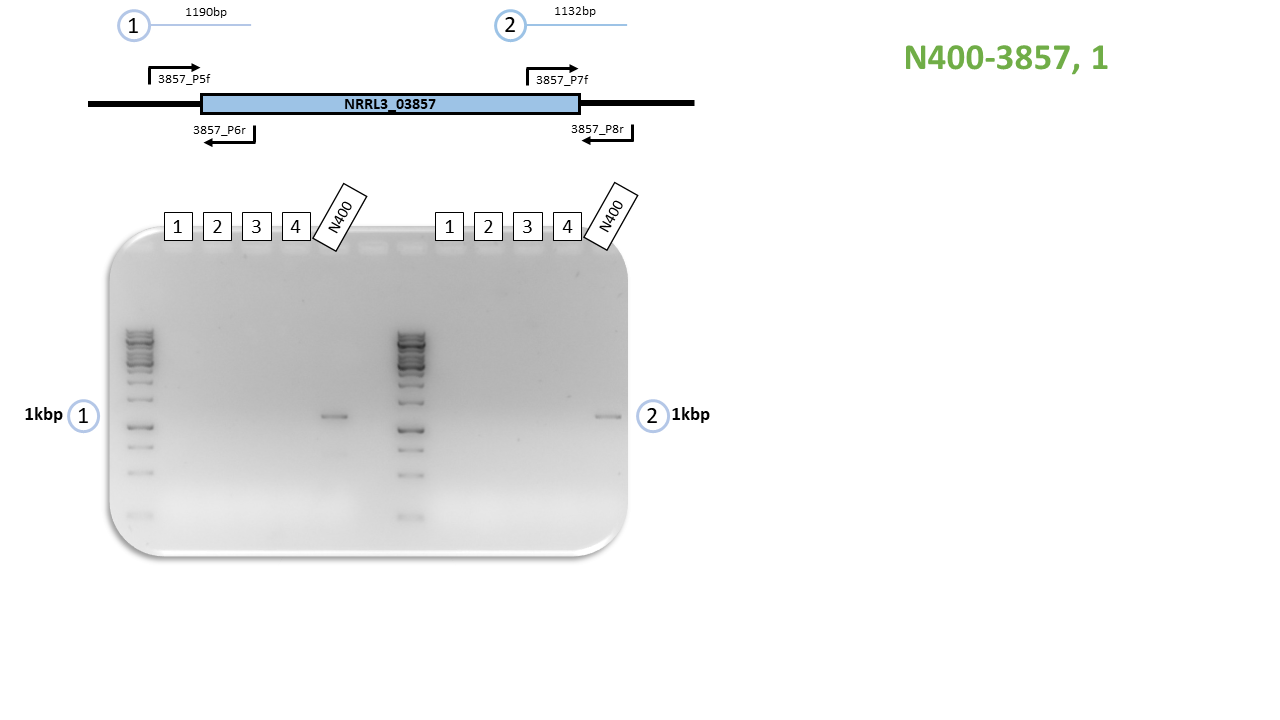


**C**

**D**
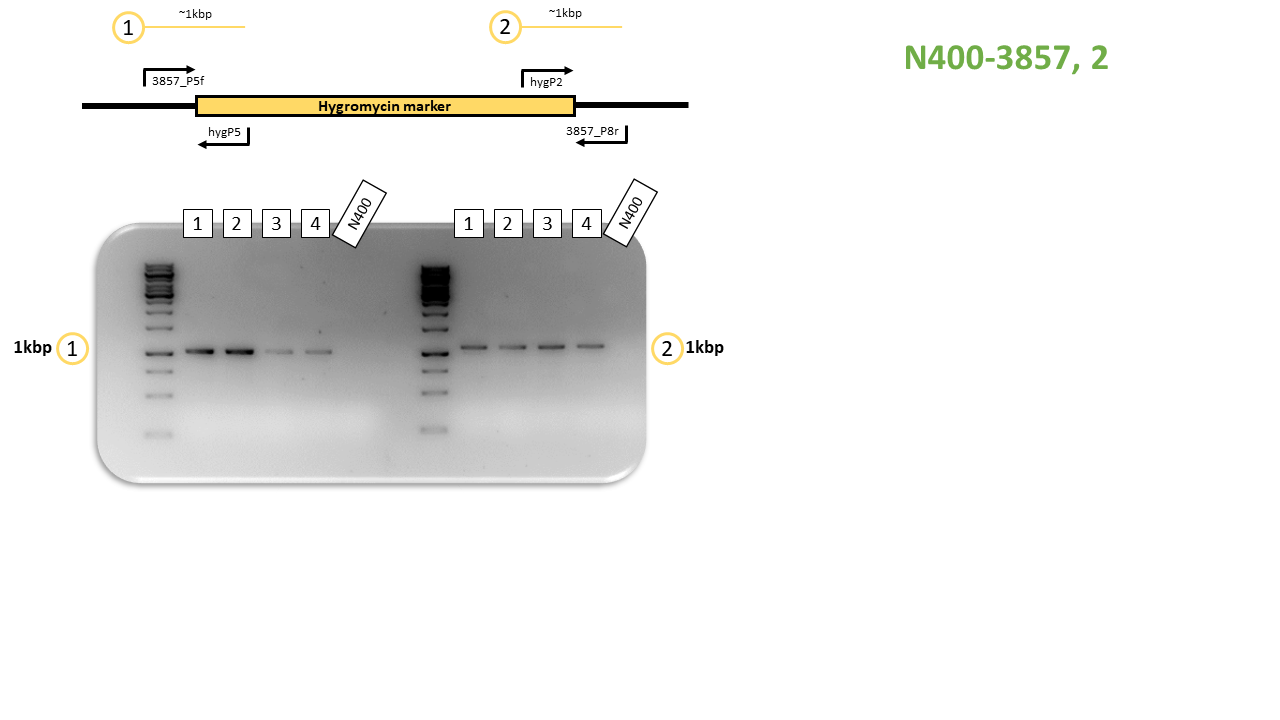


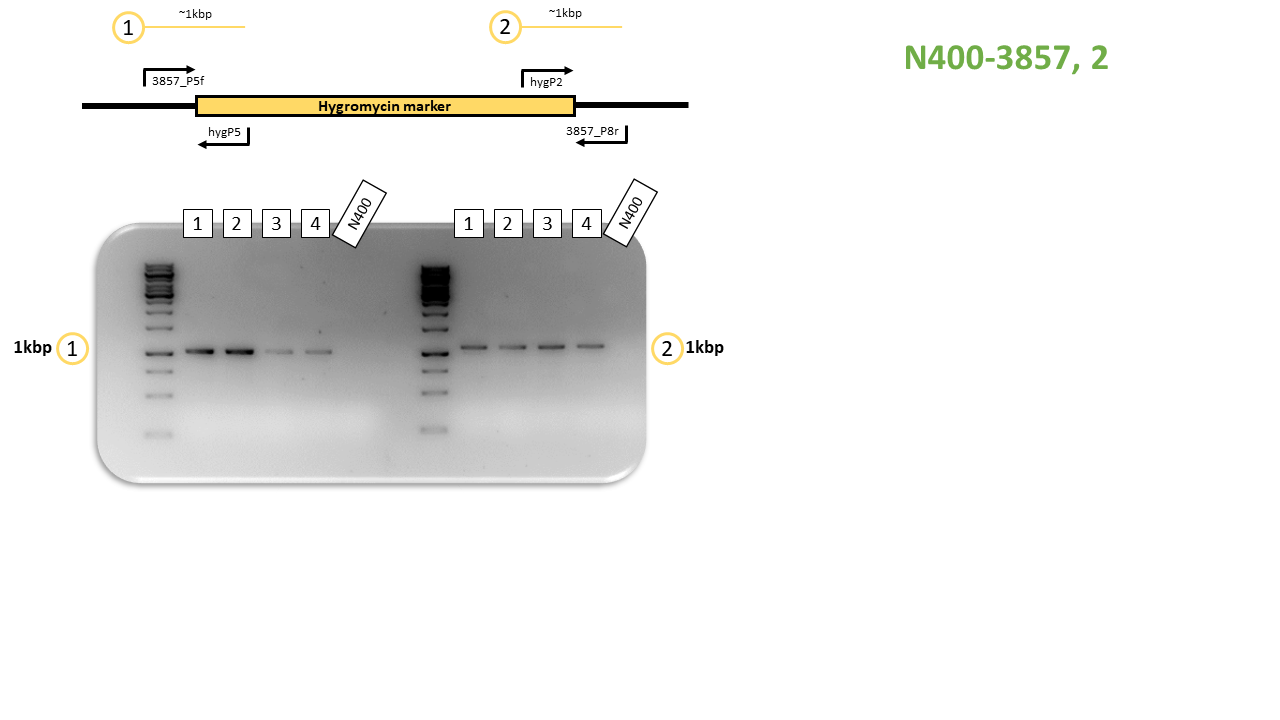


Supplemental Figure 3. Diagnostic PCR to confirm the deletion of NRRL3_03857 in MA612.27. A and C) Schematic representation of NRRL3_03847 locus before and after deletion and the sizes of the expected PCR fragments in N400 and ∆*NRRL3_03857* using the indicated primers. B and D) Agarose gel pictures of the PCR products. The position of the 1-kb molecular marker is indicated.
