# Supplementary material for: Genetic Characterization of Mutations Related to Conidiophore Stalk Length Development in Aspergillus niger Laboratory Strain N402
Source: Front Genet. 2021 Apr 20;12:666684. doi: 10.3389/fgene.2021.666684 (PMC8093798; doi:10.3389/fgene.2021.666684)
Supplement: Supplementary Figure 4 — Southern blot analysis to verify the deletion of NRRL3_06646 in N400. (A) Schematic representation of NRRL3_06646 locus in N400 and in the mutant. DNA fragments expected to hybridize with the probe after digestion of genomic DNA with BamHI are indicated. (B) Genomic DNA of putative ΔNRRL3_06646 mutants and control strain N400 was analyzed. Strain MA608.2 was selected for further studies as it displayed the expected band of the digested genomic DNA. [file Data_Sheet_4.DOCX]

Supplemental Figure 4


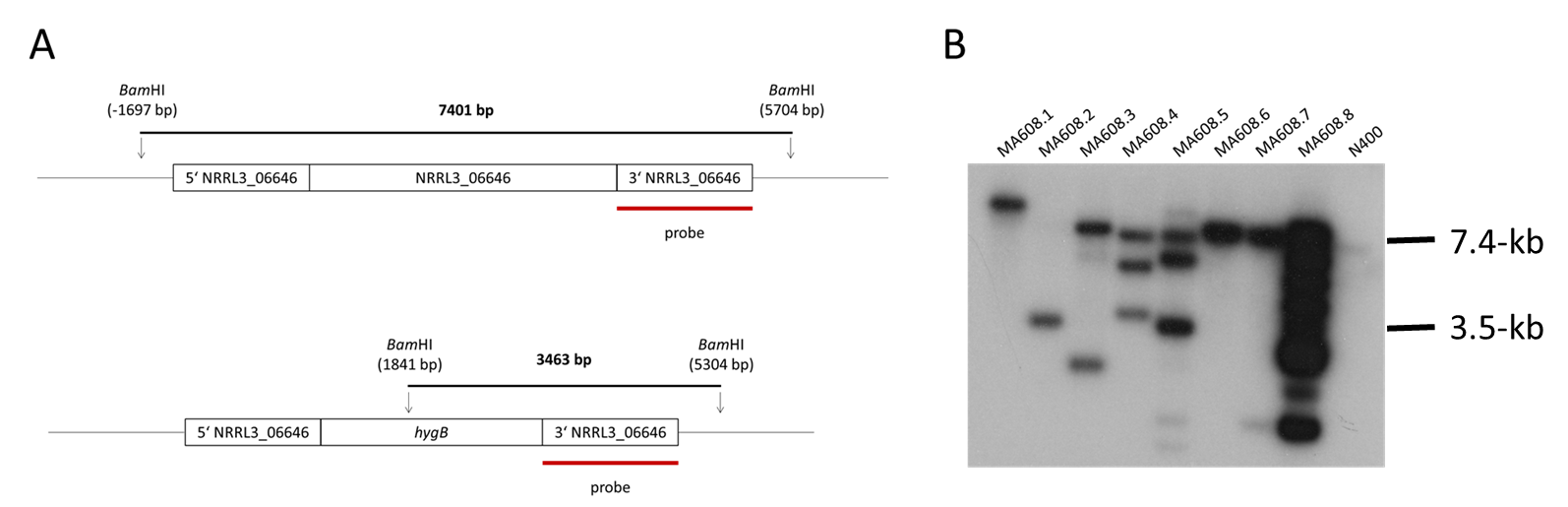


**Supplemental Figure 4**. Southern blot analysis to verify the deletion of NRRL3_06646 in N400. A) Schematic representation of NRRL3_06646 locus in N400 and in the mutant. DNA fragments expected to hybridize with the probe after digestion of genomic DNA with *Bam*HI are indicated. B) Genomic DNA of putative *∆NRRL3_06646* mutants and control strain N400 was analyzed. Strain MA608.2 was selected for further studies as it displayed the expected band of the digested genomic DNA.
