# Supplementary material for: Genetic Characterization of Mutations Related to Conidiophore Stalk Length Development in Aspergillus niger Laboratory Strain N402
Source: Front Genet. 2021 Apr 20;12:666684. doi: 10.3389/fgene.2021.666684 (PMC8093798; doi:10.3389/fgene.2021.666684)
Supplement: Supplementary Figure 5 — Diagnostic PCR to confirm the deletion of NRRL3_06646 and NRRL3_03857 in N400. (A) Schematic representation of NRRL3_03857 locus before and after deletion and the sizes of the expected PCR fragments in N400 and ΔNRRL3_03857 with the indicated primers. (B) Schematic representation of NRRL3_06646 locus before and after deletion and the sizes of the expected PCR fragments in N400 and ΔNRRL3_06646 using the indicated primers. Transformant 3 with the expected sizes of the PCR products was selected for further experiments and named BY4.1. [file Data_Sheet_5.DOCX]

Supplemental Figure 5

A B


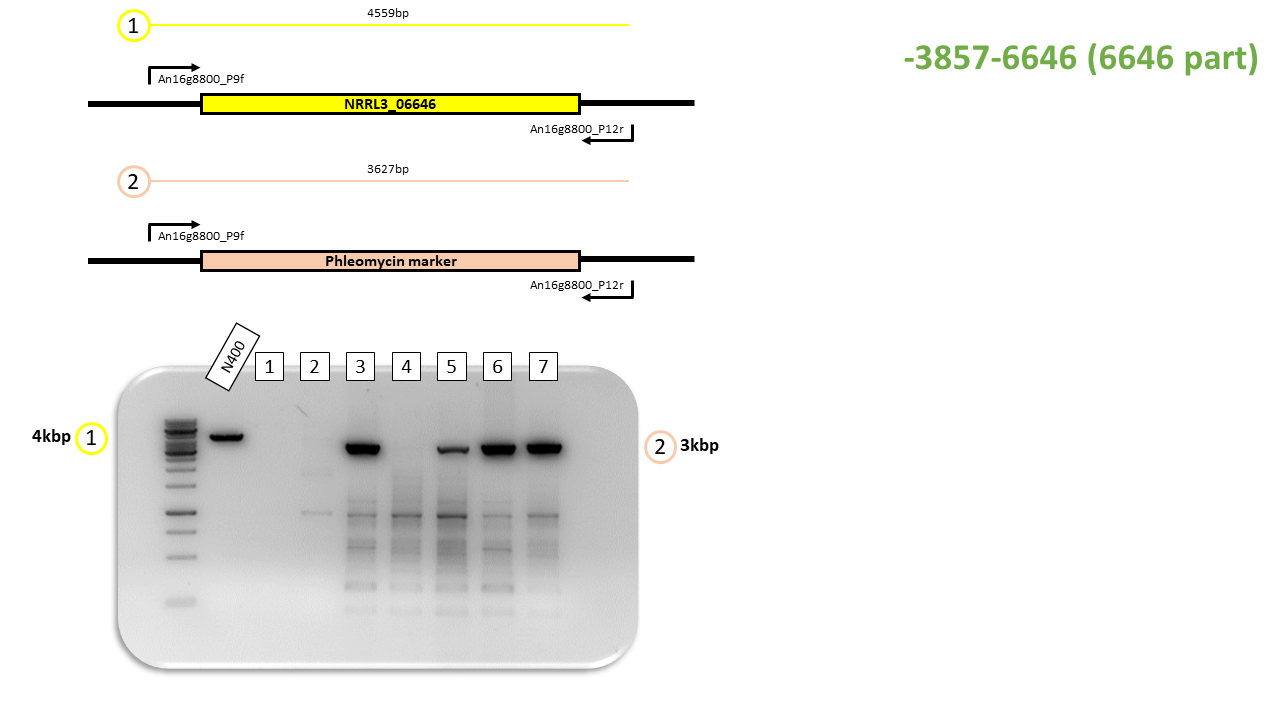

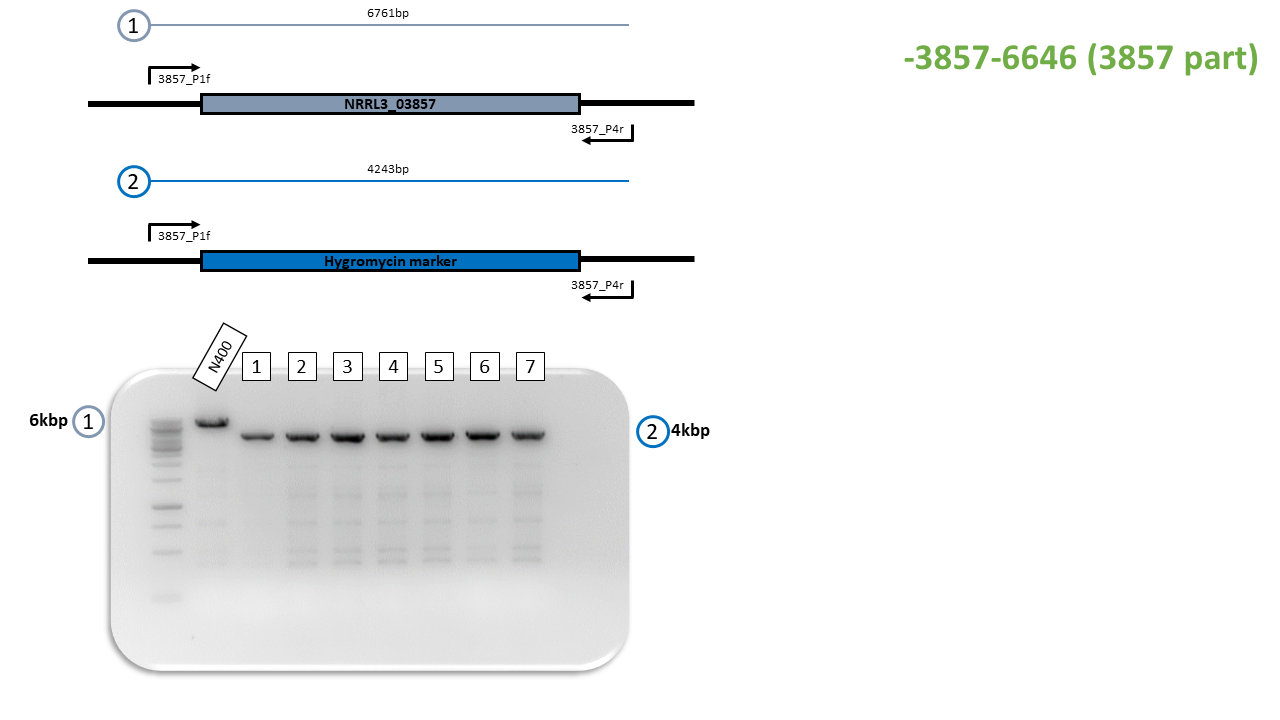


**Supplemental Figure 5.** Diagnostic PCR to confirm the deletion of NRRL3_06646 and NRRL3_03857 in N400. A) Schematic representation of NRRL3_03857 locus before and after deletion and the sizes of the expected PCR fragments in N400 and ∆*NRRL3_03857* with the indicated primers. B) Schematic representation of NRRL3_06646 locus before and after deletion and the sizes of the expected PCR fragments in N400 and ∆*NRRL3_06646* using the indicated primers. Transformant 3 with the expected sizes of the PCR products was selected for further experiments and named BY4.1.
