# Supplementary material for: Genetic Characterization of Mutations Related to Conidiophore Stalk Length Development in Aspergillus niger Laboratory Strain N402
Source: Front Genet. 2021 Apr 20;12:666684. doi: 10.3389/fgene.2021.666684 (PMC8093798; doi:10.3389/fgene.2021.666684)
Supplement: Supplementary Figure 6 — Analyses of radial growth and spore density in N400, N401, and N402. Spores of A. niger strains N400, N401, and N402 were point inoculated (10,000 spores/5 μl) and grown for 7 days at 30°C on plates containing MM with glucose before measuring the colony diameter and harvesting and counting the spores. Means represented with different number of stars are significantly different (Student’s t test P-value < 0.05). [file Data_Sheet_6.DOCX]

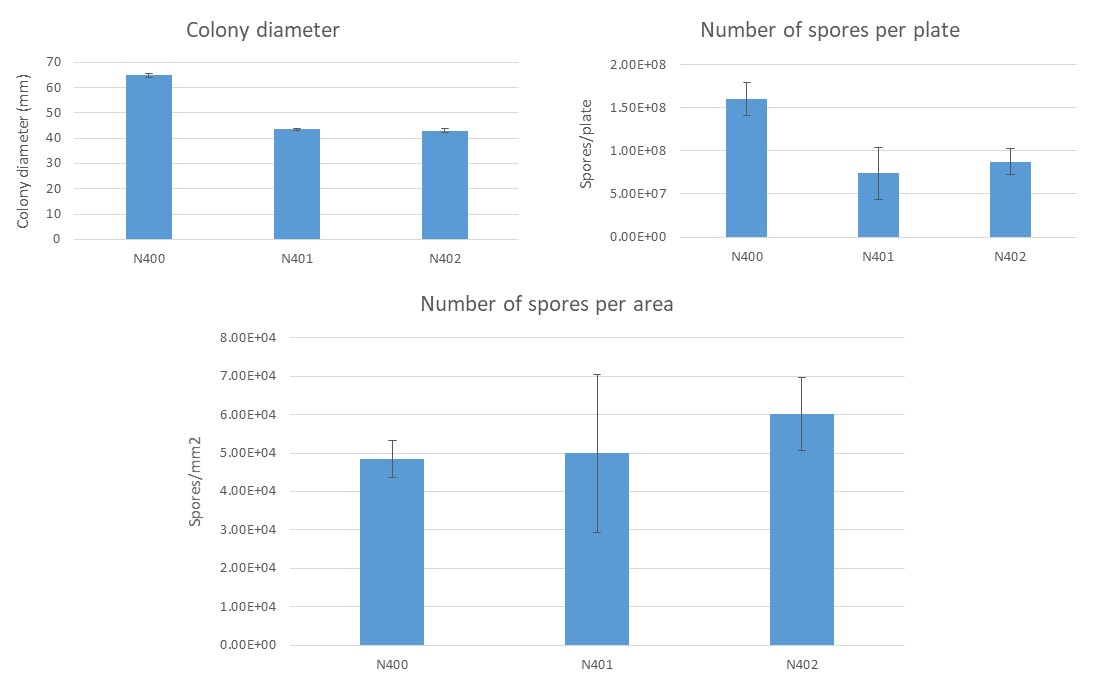


**Supplemental Figure 6.** Analyses of radial growth and spore density in N400, N401 and N402. Spores of *A. niger* strains N400, N401 and N402 were point inoculated (10000 spores/5μl) and grown for 7 days at 30° C on plates containing MM with glucose before measuring the colony diameter and harvesting and counting the spores. Means represented with different number of stars are significantly different (Student’s TTEST P-value<0.05).
