# Supplementary material for: Genetic Characterization of Mutations Related to Conidiophore Stalk Length Development in Aspergillus niger Laboratory Strain N402
Source: Front Genet. 2021 Apr 20;12:666684. doi: 10.3389/fgene.2021.666684 (PMC8093798; doi:10.3389/fgene.2021.666684)
Supplement: Supplementary Figure 7 — Phenotypic analysis of A. niger strain N400 and its derivatives (see Table 1 for details regarding the strains). (A) Radial growth diameter measurements and (B) Conidiophore stalk length measurements of N400, N401, and N402, mutants with targeted deletion of cspA (ΔNRRL3_03857), cspB (ΔNRRL3_06646), and cspA and cspB (ΔNRRL3_03857 ΔNRRL3_06646), and representative segregants. Means represented with different letters are significantly different (Tukey’s HSD, P-value < 0.001). [file Data_Sheet_7.DOCX]

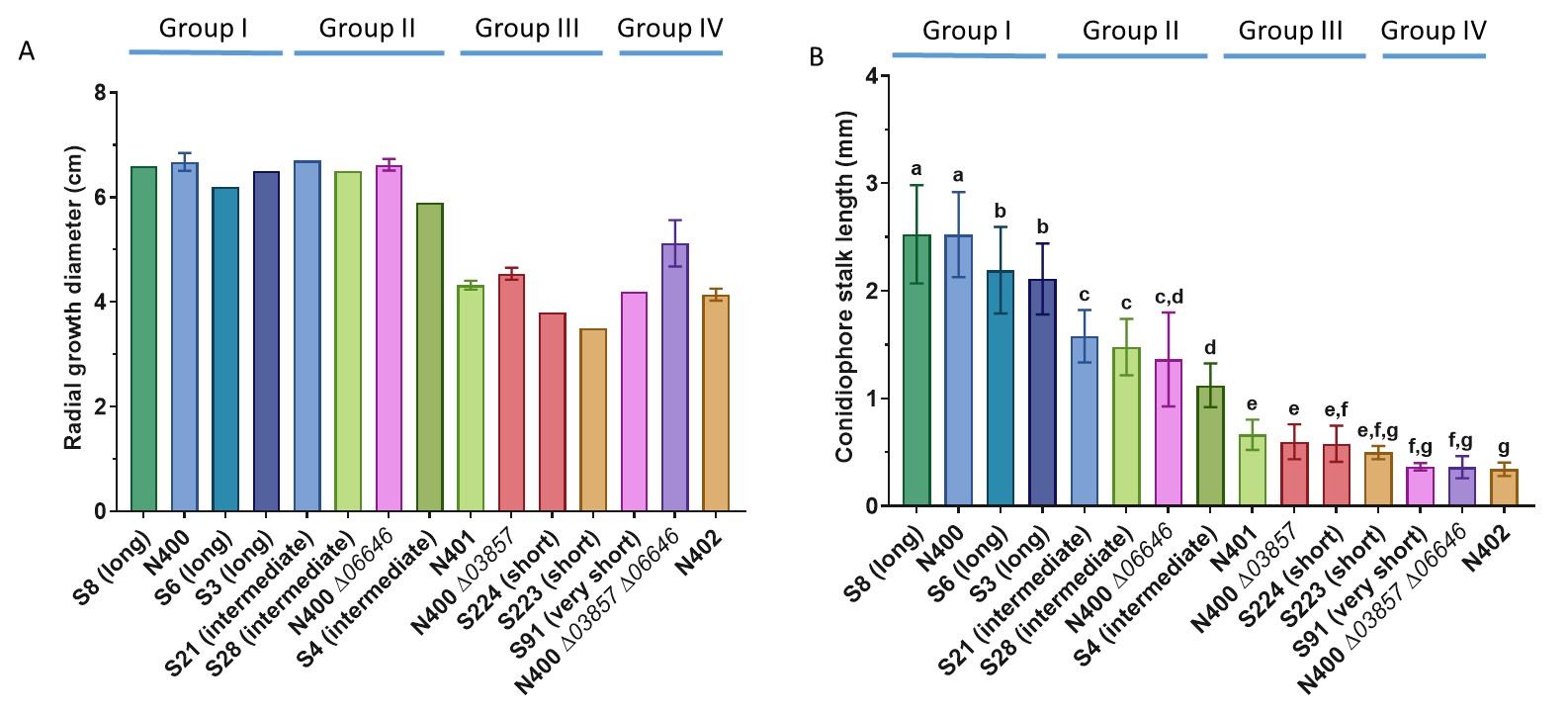


**Supplemental Figure 7.** Phenotypic analysis of *A. niger* strain N400 and its derivatives (See Table 1 for details regarding the strains). A) Radial growth diameter measurements and B) Conidiophore stalk length measurements of N400, N401, and N402, mutants with targeted deletion of *cspA* (*∆NRRL3_03857*), *cspB* (*∆NRRL3_06646*) and *cspA* and *cspB* *(∆NRRL3_03857 ∆NRRL3_06646*), and representative segregants. Means represented with different letters are significantly different (Tukey's HSD, P-value<0.001).
