# Supplementary material for: Genetic Characterization of Mutations Related to Conidiophore Stalk Length Development in Aspergillus niger Laboratory Strain N402
Source: Front Genet. 2021 Apr 20;12:666684. doi: 10.3389/fgene.2021.666684 (PMC8093798; doi:10.3389/fgene.2021.666684)
Supplement: Supplementary Figure 8 — Distribution of conidiophore stalk lengths of the segregants having a specific phenotype in comparison to that of all 221 segregants. [file Data_Sheet_8.DOCX]

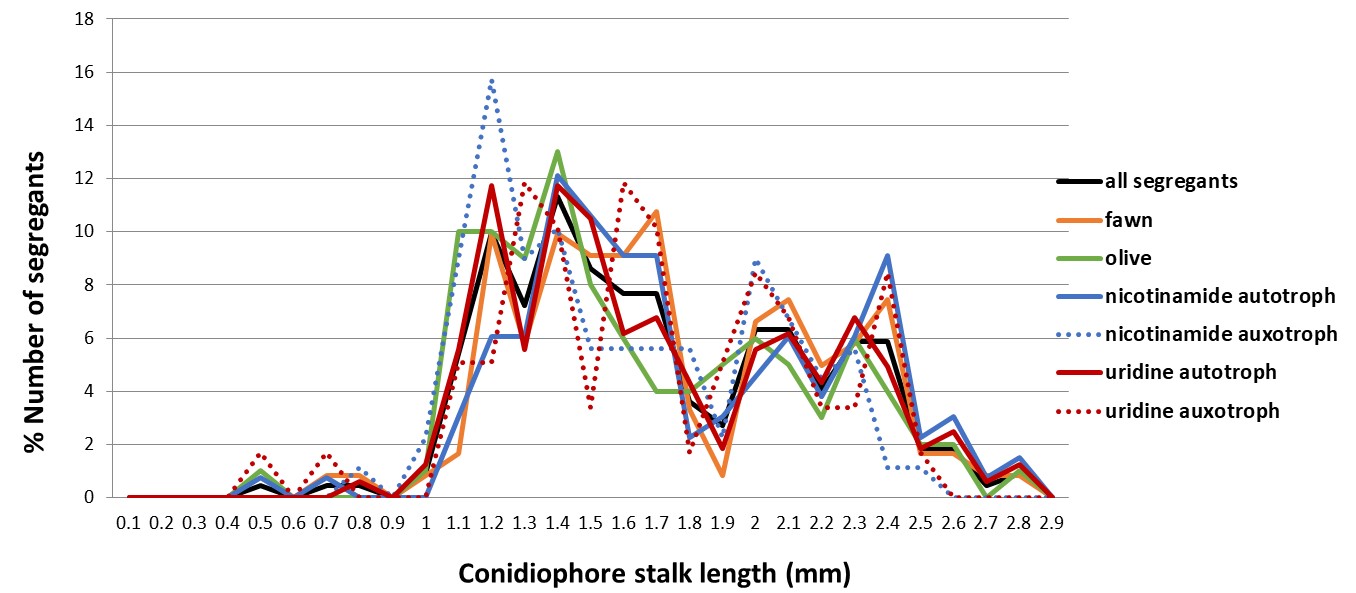


**Supplemental Figure 8.** Distribution of conidiophore stalk lengths of the segregants having a specific phenotype in comparison to that of all 221 segregants.
