# Supplementary material for: Genetic Characterization of Mutations Related to Conidiophore Stalk Length Development in Aspergillus niger Laboratory Strain N402
Source: Front Genet. 2021 Apr 20;12:666684. doi: 10.3389/fgene.2021.666684 (PMC8093798; doi:10.3389/fgene.2021.666684)
Supplement: Supplementary Table 1 — Primers used in this study. [file Table_1.DOCX]

Supplemental Table 1. Primers used in this study

| **Primer name** | **Sequence 5’-3** | **Used for** |
| --- | --- | --- |
| PksAP1f | CGAGAACCGGGTAGATATGGC | Deletion of pksA in N400; amplification of pksA-hygB 5’; |
| PksAP4r | CAGATTCAGCTCCGAAATGCC | Deletion of pksA in N400; amplification of pksA-hygB 5’; |
| HygP8f | AAAGTTCGACAGCGTCTCC | Deletion of pksA in N400; amplification of pksA-hygB 3’; |
| HygP9r | GGCGTCGGTTTCCACTATC | Deletion of pksA in N400; amplification of pksA-hygB 3’; |
| ku70P1NotI | AAGGAAAAAAGCGGCCGCCAGAACGGCTTGATGACGG | Amplification of kusA::amdS disruption construct |
| ku70P4KpnI | GGGGTACCGGCGCTTGCCTTCGTAAGA | Amplification of kusA::amdS disruption construct |
| 9kb_gRNA_Fw1 | CTGTTTAACCTGGCCATTCGGTTTTAGAGCTAGAAATAGCAAG | Construction on pBY1.1 |
| 9kb_gRNA_Rv1 | CGAATGGCCAGGTTAAACAGGACGAGCTTACTCGTTTCGT | Construction on pBY1.1 |
| pTE1_Fw | CCTTAATTAAACTCCGCCGAACGTACTG | Construction on pBY1.1 |
| pTE1_Rv | CCTTAATTAAAAAAGCAAAAAAGGAAGGTACAAAAAAGC | Construction on pBY1.1 |
| 3857_P1f | ACTACCAACACACTGGCCCTG | Amplifying 3’ flank of NRRL3_03857 |
| 3857_P2r | CAATTCCAGCAGCGGCTTGGTTGCTTCCTCCCCATACAC | Amplifying 3’ flank of NRRL3_03857 |
| 3857_P3f | ACACGGCACAATTATCCATCGAGTTCATGTCTGCCTTTACAGC | Amplifying 5’ flank of NRRL3_03857 |
| 3857_P4r | ATGCCTGCGAGCCTTCTGT | Amplifying 5’ flank of NRRL3_03857 |
| HygP6f | AAGCCGCTGCTGGAATTGGGCTCTGAGGTGCAGTGGAT | Amplification of hygB 5’ fragment |
| HygP9r | GGCGTCGGTTTCCACTATC | Amplification of hygB 5’ fragment |
| HygP7r | CGATGGATAATTGTGCCGTGTTGGGTGTTACGGAGCATTCA | Amplification of hygB 3’ fragment |
| HygP8f | AAAGTTCGACAGCGTCTCC | Amplification of hygB 3’ fragment |
| 3857_P5f | GGAGAGCGGACCAGGCTATAA | Diagnostic PCR 5’ flank |
| 3857_P6r | GGAAAGGACGGTTTGGTTGTC | Diagnostic PCR 5’ flank |
| 3857_P7f | GGCCCAGAAAGTGGAATGG | Diagnostic PCR 3’ flank |
| 3857_P8r | TCTGCGATGATCAGTGCATGT | Diagnostic PCR 3’ flank |
| HygP2f | CATGCATGGTTGCCTAGTGAA | Diagnostic PCR 3’ flank |
| HygP5r | ATCCACTGCACCTCAGAGCC | Diagnostic PCR 5’ flank |
| An16g08800_P10r | CAATTCCAGCAGCGGCTTGATGTGTGTGATAATGATAGGA | Amplifying 5’ flank of NRRL3_06646 |
| An16g08800_P11f | ACACGGCACAATTATCCATCGCTTGGCACGGACCAAGCAGTG | Amplifying 3’ flank of NRRL3_06646 |
| An16g08800_P12r | AAGGCGTAAGTGCATCCAGC | Amplifying 3’ flank of NRRL3_06646 and diagnostic PCR |
| An16g08800_P9f | GTCCCTCACCTCGACACGTT | Amplifying 5’ flank of NRRL3_06646 and diagnostic PCR |
| PhleoP4f | AAGCCGCTGCTGGAATTGCTCTTTCTGGCATGCGGAG | Amplification of phleo 5’ fragment |
| PhleoP8r | GGAAGTTCGTGGACACGACC | Amplification of phleo 5’ fragment |
| PhleoP6f | AAGTTGACCAGTGCCGTTCC | Amplification of phleo 3’ fragment |
| PhleoP5r | CGATGGATAATTGTGCCGTGTGGAGCATTCACTAGGCAACCA | Amplification of phleo 3’ fragment |
