# Supplementary material for: Genetic Characterization of Mutations Related to Conidiophore Stalk Length Development in Aspergillus niger Laboratory Strain N402
Source: Front Genet. 2021 Apr 20;12:666684. doi: 10.3389/fgene.2021.666684 (PMC8093798; doi:10.3389/fgene.2021.666684)
Supplement: Supplementary Table 2 — Results of one-way ANOVA of the conidiophore stalk lengths of different strains used in this study. [file Table_2.DOCX]

Supplemental Table 2. Results of one-way ANOVA of the conidiophore stalk lengths of different strains used in this study

| **Tukey's multiple comparisons test** | **Mean difference** | **95.00% CI of difference** | **Significant?** | **Adjusted P-value** |
| --- | --- | --- | --- | --- |
| N400 vs. N401 | 1.861 | 1.661 to 2.062 | Yes | <0.0001 |
| N400 vs. N402 | 2.183 | 1.988 to 2.377 | Yes | <0.0001 |
| N401 vs. N402 | 0.3214 | 0.1128 to 0.5301 | Yes | 0.0002 |
| N400 vs. N400 Δ03857 | 1.927 | 1.766 to 2.088 | Yes | <0.0001 |
| N401 vs. N400 Δ03857 | 0.0653 | -0.1127 to 0.2433 | No | 0.9299 |
| N402 vs. N400 Δ03857 | -0.2561 | -0.4276 to -0.08467 | Yes | 0.0003 |
| N400 vs. N400 Δ06646 | 1.161 | 0.9691 to 1.353 | Yes | <0.0001 |
| N401 vs. N400 Δ06646 | -0.7003 | -0.9066 to -0.494 | Yes | <0.0001 |
| N402 vs. N400 Δ06646 | -1.022 | -1.222 to -0.8211 | Yes | <0.0001 |
| N400 vs. N400 Δ03857 Δ06646 | 2.163 | 2.005 to 2.321 | Yes | <0.0001 |
| N401 vs. N400 Δ03857 Δ06646 | 0.3018 | 0.1266 to 0.477 | Yes | <0.0001 |
| N402 vs. N400 Δ03857 Δ06646 | -0.0196 | -0.1881 to 0.1489 | No | 0.9999 |
| N400 Δ03857 vs. N400 Δ06646 | -0.7656 | -0.9342 to -0.597 | Yes | <0.0001 |
| N400 Δ03857 vs. N400 Δ03857 Δ06646 | 0.2365 | 0.1079 to 0.3652 | Yes | <0.0001 |
| N400 vs. N400 Δ9kb | 2.089 | 1.921 to 2.257 | Yes | <0.0001 |
| N400 Δ06646 vs. N400 Δ03857 Δ06646 | 1.002 | 0.8365 to 1.168 | Yes | <0.0001 |
